# Discharge Readiness Among Primary Caregivers in Pediatric Medical–Surgical Units in Jeddah, Saudi Arabia

**DOI:** 10.3390/children11121447

**Published:** 2024-11-27

**Authors:** Maha A. Alzahrani, Manal F. Alharbi

**Affiliations:** 1 Collage of Nursing, King Saud University, Riyadh 11451, Saudi Arabia; 2 Maternal and Child Health Nursing Department, Faculty of Nursing, King Abdulaziz University, Jeddah 22252, Saudi Arabia; 3 Maternal & Child Health Nursing Department, College of Nursing, King Saud University, Riyadh 12372, Saudi Arabia; maalwahbi@ksu.edu.sa

**Keywords:** humans, child, patient discharge, discharge readiness, caregivers, hospitals, Saudi Arabia, regression analysis

## Abstract

**Background/Objectives:** Preparing families to support children after hospital discharge is crucial, particularly due to the fragile health of pediatric patients and the care required at home. In this study, the aim was to assess the readiness for hospital discharge among primary caregivers of pediatric patients in medical–surgical units in Jeddah, Saudi Arabia, and to identify factors influencing their preparedness. **Methods:** A quantitative cross-sectional study was conducted among 258 primary caregivers recruited from two hospitals in Jeddah: King Abdulaziz University Hospital (KAUH) and a Ministry of Health (MOH) hospital. A purposive sampling method was used. Data were collected through the Pediatric Readiness for Hospital Discharge Scale (Ped-RHDS) and the Quality of Discharge Teaching Scale (QDTS), translated into Arabic. Descriptive statistics, t-tests, and multiple regression analyses were employed to identify key predictors of discharge readiness. **Results:** Caregivers reported moderate to high readiness for discharge, with mean scores of 8.28 (SD = 2.65) for personal strength and 8.62 (SD = 2.26) for their child’s strength. Knowledge scores averaged 7.49 (SD = 3.27). The quality of discharge teaching was higher at KAUH (M = 6.43, SD = 2.56) than at the MOH hospital (M = 5.48, SD = 2.89, *p* = 0.006). Caregiver age, child age, and discharge teaching quality were significant predictors of readiness (*p* < 0.05). **Conclusions:** In this study, the importance of discharge readiness is emphasized, highlighting the role of discharge education in enhancing preparedness. Addressing caregivers’ specific needs, especially for younger children or prolonged stays, can improve readiness and reduce post-discharge complications.

## 1. Introduction

Preparing families to support their children after hospital discharge is complex and intricate. Research on preparedness for discharge is gaining importance due to the fragile health of pediatric patients and the elevated level of care required at home [1]. More than one-fifth of caregivers encounter readiness difficulties when transitioning their child’s care from the hospital to the home [2]. An estimated one out of five families experience a readmission or emergency room visit due to a failure to comprehend discharge instructions [3]. The transition from hospital to home-based care is greatly influenced by the readiness of caregivers for discharge, which is particularly true for primary caregivers of children who often face significant challenges in providing care after discharge [4]. The term “primary caregiver” is defined as “the individual who provides the greatest amount of care to and accepts responsibility for the sick child during the illness, treatment, and rehabilitation process” [5]. Therefore, caregivers need support in helping children adjust well when transitioning to home.

Patient readiness for hospital discharge (RHD) was defined by Fenwick [6] as an ability to cope with the realities of life after hospitalization. Discharge readiness is an outcome indication of proper hospital care [7]. According to a study conducted by Berry et al. [8], parental preparedness for discharge correlates with the incidence of unplanned 30-day readmissions. Assessing discharge readiness is crucial for improving patient safety, satisfaction, and outcomes during the transition from hospital- to home-based recovery and care [9,10]. As per the analysis by Galvin et al. [11], readiness for hospital discharge has the four following dimensions: (a) functional ability, which involves the capability to take care of oneself at home; (b) having appropriate support in place to assist with post-hospital tasks; (c) being psychologically prepared to handle the process of change; and (d) knowing how to manage their own care and address minor issues that may arise after leaving the hospital. The readiness for discharge of the caregiver involves objectively evaluating the patient’s capacity to recover post-discharge, as well as considering the caregiver’s personal perception of the readiness for discharge [12]. The perceptions of patients and their families regarding readiness serve as crucial indicators of the discharge preparation process. These perceptions offer insight into the potential risks associated with a challenging post-discharge transition, which may result in adverse outcomes and readmission [4].

Nurses play a crucial role in preparing the family before hospital discharge; discharge planning is critical to completing the patient’s recovery process [13,14]. Implementing an assessment that identifies patients with low readiness for hospital discharge can assist nurses and other healthcare professionals improve patient readiness and prevent problematic discharges [15]. The primary approach employed by nurses for the preparation of patients and their families for discharge and the subsequent transition home from the hospital is through the provision of comprehensive education and instruction [16].

Several factors may affect caregivers’ readiness for discharge from a hospital setting to the home, such as satisfaction with hospitalization experience [17], quality of discharge education [18], and age-related vulnerabilities [19]. In contrast to clinical criteria for assessing readiness, the authors of [20] contend that the quality of discharge education largely predicts discharge readiness. Effective discharge teaching significantly improves caregivers’ capabilities, reduces the ambiguity surrounding their child’s illness, and enhances their confidence in being prepared for discharge [21]. Previous studies have highlighted the unique challenges and needs specific to these populations, emphasizing the importance of tailored discharge planning to ensure caregivers are adequately prepared for the transition to home care. However, there is a notable lack of comprehensive data that provide an overview of caregiver experiences in general pediatric hospitalizations, leaving a gap in understanding the broader scope of discharge readiness across more diverse pediatric patient populations [22].

There are evident adverse outcomes related to low perceptions of discharge readiness; additionally, the quality of discharge education plays a significant role in supporting the family transition home after hospitalization. To our knowledge, there is a limited number of studies to support pediatric patients and their caregivers transition post-discharge. In Saudi Arabia, the research concerning discharge readiness has not yet been implemented.

## 2. Materials and Methods

### 2.1. Theoretical Framework

**Figure 1:**
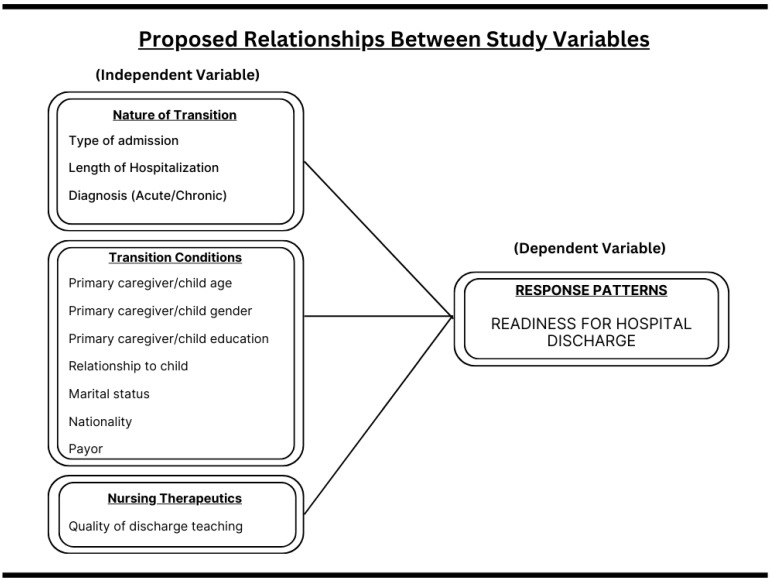
Hypothesized theoretical framework.

Meleis’ middle-range theory of transition will be utilized as a conceptual framework for examining the transition for pediatric primary caregivers during discharge from a hospital to a patient’s home. This middle-range theory aims to investigate, understand, and forecast the experiences of individuals during various kinds of transitions, including transitions related to health and illness, situational changes, personal growth, and organizational shifts. Based on Meleis [23], transitions theory consists of the following major concepts: types and patterns of transitions, properties of transition experiences, transition conditions (facilitators and inhibitors), patterns of response/process and outcome indicators, and nursing therapeutics. Figure 1 illustrates the hypothesized theoretical framework.

### 2.2. Research Design

In this study, a quantitative cross-sectional design was used to examine phenomena or relationships at a specific time [24].

### 2.3. Target Population

The target population consisted of the primary caregivers of pediatric patients admitted to the medical and surgical pediatric units at a university hospital and a governmental hospital under the Ministry of Health (MOH) in Jeddah, Saudi Arabia. These caregivers were responsible for providing care during hospitalization and after discharge. They were recruited from pediatric inpatient units.

### 2.4. Sampling

Sample size determination is critical in research planning [25]. The sample size was calculated using G*Power 3.1 [26]. A two-tailed test with an effect size of 0.15, 5% significance level, and 0.95 power yielded a minimum required sample size of 214. To account for potential missing data, 20% more participants were added, resulting in a total sample size of 257 [27].

### 2.5. Sampling Design

A purposive (non-probability sampling) was used to recruit 257 primary caregivers from medical and surgical pediatric units. Purposive sampling involves deliberately selecting individuals with unique characteristics or experiences relevant to the study [28]. While this method is cost-effective and widely used, it has the drawback of potential researcher bias in the selection process [29,30].

### 2.6. Inclusion and Exclusion Criteria

The sample consisted of primary caregivers of hospitalized children from King Abdulaziz University Hospital (KAUH) and a Ministry of Health (MOH) hospital in Jeddah, Saudi Arabia. Inclusion criteria were as follows: (a) caregivers aged 18 or older with a child aged 18 or younger; (b) discharge to the home setting; (c) primary responsibility for the child’s care after discharge; and (d) sufficient Arabic language skills for consent and study participation. Caregivers of children discharged to hospice care and transferred to extended care were excluded.

### 2.7. Procedure of Data Collection

Daily discharge lists were obtained from discharge instructors and in-charge nurses at the KAUH and MOH hospitals. Caregivers were recruited within four hours of discharge after the principal investigator introduced this study. A demographic survey, Ped-RHDS, and QDTS data were collected from eligible participants. Consent was obtained, and caregivers completed electronic forms on iPads/tablets to facilitate data collection and analysis. Ethical approval was secured from the KAUH and MOH hospitals’ committees, and the principal investigator personally handled data collection with the help of trained nursing interns.

### 2.8. Variables and Their Measurement

Three instruments were used, a Demographic questionnaire, the Quality of Discharge Teaching Scale (QDTS), and the Readiness for Hospital Discharge Scale (RHDS).

#### 2.8.1. Demographic Questionnaire

A 10-question survey gathered demographic information divided into (a) nature of transition (admission type, diagnosis, length of stay) and (b) transition conditions (caregiver/child age, caregiver gender, relationship to child, marital status, nationality, payor, and education level).

#### 2.8.2. Readiness for Hospital Discharge Scale (Ped-RHDS)

The Ped-RHDS measures caregivers’ readiness for pediatric discharge, assessing knowledge for post-discharge care, personal and emotional status, coping ability, and expected support [20]. In this study, the short form (8 items) was used to reduce survey fatigue, which can affect response accuracy [31]. The Ped-RHDS is scored on a 10-point Likert scale (0 to 10), with higher scores indicating greater discharge readiness. The Cronbach’s alpha for the Ped-RHDS is 0.73, confirming its reliability [4].

#### 2.8.3. Quality of Discharge Teaching Scale (QDTS)

The QDTS, developed by Dr. Weiss, evaluates the effectiveness of discharge teaching [32]. It consists of 19 items across the following three subscales: needed content, received content, and delivered content. Responses are measured on a 10-point Likert scale. The tool is highly reliable, with a Cronbach’s alpha of 0.88 for caregivers of hospitalized children [33].

These instruments assessed the independent variable (quality of discharge teaching, 19 items) and the dependent variable (readiness for hospital discharge, 8 items), measured at the interval level. Demographic data, measured at nominal and ordinal levels, were also included to explore their influence on the study outcomes. This comprehensive approach aims to enhance healthcare practices for patient discharge and inform policymakers to improve patient outcomes.

### 2.9. Tool Translation

Following Weiss et al. [20], and a thorough literature review, the original QDTS and Ped-RHDS tools were developed in English. A rigorous translation process, including forward and back translation, was employed to ensure accuracy [28]. Independent translators handled both stages. Permission for translation was obtained from Dr. Weiss. The translation from English to Arabic involved four Ph.D.-qualified experts proficient in both languages. Two experts conducted the forward translation, followed by a back translation by another two experts. This approach ensured the target version remained faithful to the original text [34]. Dr. Weiss supervised the process, providing guidance and feedback at each stage to ensure accuracy and consistency. The translation team included Dr Adel Bashatah (King Saud University), Dr Sabria Johar (King Saud bin Abdulaziz University for Health Sciences), and Dr Rasha AlSaiegh and Dr Salmah Alghamdi (King Abdulaziz University). Their expertise in nursing and language studies and commitment contributed to the high-quality final translation.

### 2.10. Pilot Study

Performing a pilot test is essential in identifying potential issues before initiating the final study and determining feasibility. A pilot study was conducted with 30 participants, representing 10% of the sample size, to test research protocols, data collection instruments, and recruitment strategies [28]. The pilot evaluated the feasibility, duration, and design of this study, as well as the applicability of the translated tool. The pilot study was conducted from 2 November 2023 to 15 January 2024. Participants in the pilot study were excluded from the main research to ensure the validity of the results.

## 3. Results

### 3.1. Preparation for Data Analysis

Data preparation was essential for the statistical analysis. SPSS (versions 26 and 28) was used for its reliability and established use in quantitative analysis. Demographic data, such as admission type, diagnosis, and caregiver details, were collected via a questionnaire. The raw data were exported from Google Forms, organized in Excel, and imported into SPSS for analysis.

Two key instruments, the Pediatric Readiness for Hospital Discharge Scale (Ped-RHDS) and the Quality of Discharge Teaching Scale (QDTS), both used a 10-point Likert scale. Higher scores indicated greater discharge readiness or higher perceived quality. The 10-point scale improved construct validity [35], and the Likert scales with five or more options were suitable for parametric analysis [36]. All variables were properly labeled in SPSS, and data accuracy was verified by comparing dataset codes with original values. The absence of reverse-coded items simplified interpretation. The analysis followed five steps:

1. Descriptive statistics were used to describe demographic characteristics and readiness for discharge.
2. Overall readiness was defined by the mean of the items in each category to provide an average measure of responses.
3. Assumption checks were conducted to ensure the validity of each statistical test.
4. Multiple regression analysis predicted the relationship between readiness for discharge (dependent variable) and other factors (independent variables) using a stepwise selection method. F-statistics and p-values (≤0.05) determined the best model fit and significant variables [37].
5. T-tests compared the means between hospitals for overall readiness and quality of discharge, assessing statistical significance [38].

### 3.2. Data Analysis

#### 3.2.1. Demographic and Hospitalization Characteristics

A total of 258 pediatric primary caregivers participated in this study. Most admissions (86.0%) occurred through the emergency department, with 8.1% through clinics and 5.8% being planned admissions. Regarding diagnosis, 47.7% of the children had acute conditions, 45.3% had chronic conditions, and 7.0% were admitted for planned operations. The majority of caregivers were female (98.1%), with mothers accounting for 93.4% of the sample. The average caregiver age was 34.04 years (SD = 7.6), and the majority of children were either in childhood (54.7%) or infancy (34.9%). The average length of hospital stay was 7.72 days (SD = 8.5). Regarding education, 42.2% of caregivers had a bachelor’s degree, 26.0% had completed high school, and 15.1% had a middle school education. A small percentage (6.2%) were illiterate, and only 2.3% had higher education beyond a bachelor’s degree. The majority of caregivers were married (89.9%) and Saudi nationals (69.0%). Most children (77.1%) were covered by government health programs, 12.4% by charitable organizations, 6.2% paid in cash, and 4.3% used medical insurance (Table 1).

#### 3.2.2. Readiness for Hospital Discharge (RHD)

Caregivers reported relatively high levels of readiness for hospital discharge (RHD). Caregivers rated their strength on the day of discharge with a mean score of 8.28 (SD = 2.65), while the strength of their children was rated slightly higher (M = 8.62, SD = 2.26). In terms of knowledge, caregivers reported a moderate understanding of post-discharge issues (M = 7.49, SD = 3.27) and what their child was allowed to do (M = 8.04, SD = 2.98). Confidence in handling the demands of home care was high, particularly for performing medical treatments (M = 9.14, SD = 1.87), while expected support after discharge was rated moderately (M = 7.69, SD = 2.96) (Table 2).

#### 3.2.3. Quality of Discharge Teaching (QDT)

The quality of discharge teaching (QDT) provided by nurses was also rated moderately by caregivers. The overall need for information was scored at 5.96 (SD = 2.77), while the information received from nurses was rated slightly lower at 5.62 (SD = 3.18). Caregivers found that nurses were responsive to their concerns (M = 6.86, SD = 3.64) and provided consistent information across healthcare professionals (M = 7.87, SD = 3.19). Confidence in knowing how to handle emergencies was rated relatively high (M = 7.48, SD = 3.24) (Table 3).

#### 3.2.4. RHD and QTD Comparison

When comparing the two hospital types, there was no significant difference in overall readiness for discharge between the King Abdulaziz University Hospital (KAUH) and the Ministry of Health (MOH) hospital (*p* = 0.067; see Table 4). However, the quality of discharge teaching was perceived as significantly better at KAUH (M = 6.43, SD = 2.56) compared to the MOH hospital (M = 5.48, SD = 2.89, *p* = 0.006; see Table 5).

#### 3.2.5. RHD Predictors

Regression analysis identified several predictors of discharge readiness. Caregiver age and quality of discharge teaching (QDT) were significant predictors of personal readiness (F(2, 255) = 6.37, *p* = 0.002). Older caregivers and those who received comprehensive discharge education were more prepared for discharge. In terms of the child’s readiness, child age and diagnosis type significantly influenced perceptions. Caregivers of infants felt their children were less ready for discharge compared to older children (*p* = 0.013), and children with acute diagnoses were perceived as more ready for discharge than those undergoing planned operations (*p* = 0.010) (Table 6). Finally, hospital stay length negatively impacted caregivers’ coping and knowledge readiness. Longer hospital stays were associated with lower coping abilities (*p* = 0.001) and lower knowledge retention (*p* = 0.019). Caregiver nationality also influenced expectations, with non-Saudi caregivers reporting significantly lower expectations of support post-discharge (*p* < 0.001) (Table 6).

## 4. Discussion

In this study, a critical gap in research on discharge readiness in pediatric care is addressed, focusing on primary caregivers in medical and surgical departments. While discharge preparedness has gained recognition in pediatric critical care, research in non-critical care settings still needs to be expanded. This gap is significant given the growing importance of discharge preparedness in pediatric care, mainly due to children’s fragile health status and increased home care needs [1]. Our findings emphasize that caregivers in medical and surgical units face substantial barriers to feeling prepared for discharge, similar to those in critical care settings. By focusing on this underserved population, our study highlights the urgent need for tailored discharge interventions addressing the unique challenges pediatric primary caregivers face. As the first study of its kind conducted in Saudi Arabia for the pediatric population, our research provides valuable insights into caregivers’ specific needs and concerns in this cultural context, informing the development of culturally sensitive and targeted discharge preparation strategies.

### 4.1. Readiness for Hospital Discharge

In this study, it was found that discharge readiness among caregivers in pediatric medical and surgical units was moderate, addressing the first research question about the current level of discharge readiness for pediatric primary caregivers. Moderate readiness means that while many caregivers felt reasonably equipped, there were still significant challenges in managing their child’s care after leaving the hospital. These findings are consistent with the prior research indicating that although discharge readiness is generally adequate, many caregivers still require extra support and education to feel completely ready to care for their child at home [9,18,39]. Participants demonstrated moderate to high scores across all aspects of discharge readiness, including personal status, knowledge, coping, and expected support, with knowledge and expected support being the lowest-scoring components. For the low-scored aspects, simple and cost-effective interventions, such as tailored guidance or visual aids, can enhance caregivers’ preparedness by addressing weak areas. Additionally, models such as the IDEAL discharge planning framework can be employed to structure and improve the discharge process [40]. Furthermore, assessing discharge readiness (RHD) is crucial, as it is key in preventing or reducing adverse outcomes associated with discharge [41]. Utilizing an evaluation that determines patients’ readiness for leaving the hospital can help nurses and other medical staff enhance patient preparedness and avoid discharge-related issues [15]. In this study, it was also found that discharge readiness scores were similar in both organizations, indicating no significant difference in discharge readiness levels between the pediatric medical and surgical units.

### 4.2. Quality of Discharge Teaching

Despite this similarity in readiness, the quality of discharge education was notably higher at the King Abdulaziz University Hospital (KAUH) compared to the Ministry of Health Hospital (MOH). This aligns with the discharge policies of the two institutions, where KAUH emphasizes early discharge planning and comprehensive education for patients and their families, ensuring more thorough preparation. In contrast, the MOH hospital focuses more on the practical aspects of patient preparation and coordination for discharge. The stronger discharge teaching at KAUH reflects its policy focus on education, which may account for the higher quality of instruction provided to caregivers despite the similar overall readiness levels between the two hospitals. This distinction underscores the importance of robust educational support in the discharge process to improve caregiver confidence and preparedness.

In the current study at the aforementioned medical organizations, a moderate quality of discharge teaching (QDT) was reported, addressing the second question regarding the level of discharge teaching provided by nurses. The delivery sub-scale received moderate to high ratings, while the content and received sub-scales were rated at moderate levels, indicating room for improvement in these areas. This finding aligns that inconsistencies in both the content and delivery of discharge information can also negatively affect caregivers’ perceptions of discharge readiness and their overall recovery experience [42], further emphasizing the need for standardized, high-quality education during the discharge process.

### 4.3. Predictors of Readiness for Hospital Discharge

According to this study, there is a clear link between the quality of discharge teaching and higher levels of readiness for hospital discharge (RHD), specifically the personal status aspect, showing that comprehensive and empathetic education is crucial and can predict caregivers’ readiness for discharge, as they are positively correlated. This finding aligns with previous research on parents of hospitalized children, which demonstrated that nurses’ high-quality discharge teaching, particularly how information was delivered, significantly improved parental readiness for discharge [32,43]. Additionally, greater readiness was associated with fewer coping difficulties and a reduced need for post-hospitalization health services [18,32].

In addition to the quality of discharge teaching, several other predictors were identified in this study, addressing the third research question on factors influencing caregivers’ readiness for discharge. Caregiver age was positively correlated with caregiver readiness, suggesting that as the caregiver’s age increased, the personal status of the caregiver improved. This finding, which contradicts a previous study that found age-related differences causing challenges for discharge readiness, emphasizes the need for further research [44]. The earlier study specifically highlighted areas of concern like information retention and coping. Regardless, a study conducted by Ganefianty et al. [45] demonstrated a finding that aligns with the current research and reported that age was significantly linked to caregivers’ discharge readiness. Such a result indicates that age can pose challenges in some aspects, while older caregivers may express more confidence and ability due to their extended life experience or caregiver familiarity.

The child’s age was an identified predictor variable in estimating the level of readiness for discharge, and pediatric primary caregivers reported lower readiness levels when discharging infants compared with children of older ages, such as childhood or adolescence. This suggests that caregivers may feel increased apprehension or uncertainty in caring for younger children outside of the hospital environment, specifically infants. According to the study conducted by MacKay et al. [46], these parents are continuously in a grieving moment, which is stressful and tends to call for role adjustment, thus increasing their anxiety regarding how to manage care in the home setting. Holditch-Davis et al. [47] reported that caregivers of fragile babies in pediatric medical/surgical general units outside of intensive care often experience significant distress. The study highlighted the pressing need for additional support to help caregivers feel more comfortable in their caregiving role. With maturation, the caregiver is often more confident in managing the child’s needs within the comfort of their home and, thus, preparedness for discharge. Consequently, nurses bear significant importance in supporting the pediatric primary caregivers in general units, particularly those responsible for infants, through education, emotional support, and practical methods that improve caregivers’ confidence and competence to manage their infants’ health needs after discharge.

Hospital admission type strongly influences discharge readiness among pediatric primary caregivers, including acute admission, planned surgical admission, and medical admission. Caregivers of children with acute admissions demonstrated higher discharge readiness levels than children undergoing planned operations. According to Weiss et al. [18], acute cases are often uncomplicated, with fewer complications and care directives that may further emphasize a sense of control and empower caregivers to be confident in managing their child’s transition home. In contrast, caregivers whose children had undergone planned surgical procedures reported higher levels of stress and took longer to adapt and assume coping strategies, as identified by Poh et al. [48]. For these cases, critical predictors of discharge readiness included factors relating to family integration and communication, which would suggest that scheduled surgeries, by their very nature, require more prolonged support and individualized discharge planning to prepare caregivers adequately. Medical admissions did not show significant variations in readiness for discharge, demonstrating that such cases might carry different levels of complexity, neither hindering nor facilitating the transition home as much as acute or surgical cases do. It is thus important to provide personalized discharge education and support the caregivers with approaches to enhance their preparedness, especially for those patients with planned surgeries, since this can reduce readmission rates [49,50].

Interestingly, in this study, it was found that prolonged hospital stays were associated with reduced caregiver knowledge and coping ability. The caregivers of patients with long-term impairments requiring extended hospital stays often experience escalating emotional distress over time, alongside a decline in social support and difficulties in developing effective coping strategies. Research indicates that caregivers managing prolonged hospitalizations require enhanced support systems, such as psychological counseling and customized education, to improve coping skills and retain critical information effectively [51].

Moreover, the results of this study are consistent with the previous ones, pointing out that an extended period of hospitalization decreases caregivers’ knowledge and coping potential. It was documented in particular that long-term impairments of the patients with extended periods of admittance to hospitals are usually followed by a growth in the emotional stress of their caregivers, loss of social support, and an inability to develop coping measures effectively [52]. This stress may also arise due to other duties and changes in caregivers’ lives. To limit these effects, the evidence from previous research argues that increased support networks, including psychological guidance and individualized teaching, are fundamental in empowering caregivers to better cope with challenges and efficiently retain information at the prolonged duration of care [51]. Nurses are vital in guiding patients and their families through the transition process, offering personalized support, building trust, and empowering individuals to navigate their new care environment with confidence and autonomy [53]. In addition to prolonged hospital stays, the current study also determined that caregivers of other nationalities reported lower expectations of post-discharge support compared to Saudi participants. This is partially linked to the challenges faced by non-Saudi caregivers in the Saudi Arabian health system. Being from a different culture than the country of residence, navigating the healthcare system, and managing post-discharge care has become far more complex. Non-Saudi caregivers are specifically in a situation where there is a barrier with the language, unfamiliarity with local healthcare processes, and a general lack of support networks. Thus, their expectations of post-discharge care are lower than those of Saudi caregivers. As expected, the findings reflected this, where non-Saudi caregivers expressed lower confidence in receiving adequate support. It was also similarly concluded by Alotaibi et al. [54], where cultural and language differences hindered effective communication between individuals from different backgrounds, ultimately impacting the quality of care.

### 4.4. Strengths

This study is among the first on discharge readiness among pediatric caregivers in Saudi Arabia. It offers a good insight into this population, which needs to be well-researched. The use of the Pediatric Readiness for Hospital Discharge Scale and the Quality of Discharge Teaching Scale provides a comprehensive view of caregivers’ preparedness, and the quality of education received post-discharge. Additionally, comparing university and Ministry of Health hospitals adds value to the paper, while using rigorous statistical methods enhances the reliability of the findings. Moreover, the sample size was sufficient, ensuring robust and reliable results.

### 4.5. Weaknesses

Purposive sampling, upon which this study is based, may be subject to selection bias, limiting generalization to the broader population. The cross-sectional design captures data at one point, preventing analysis of outcomes such as readmissions. Cultural and language barriers might influence responses from non-Saudi caregivers. Lastly, self-reported data could introduce response bias, as caregivers may overestimate their preparedness or satisfaction with discharge teaching.

### 4.6. Future Recommendations

In light of these limitations, and as a way forward for future studies, we propose the following: Future studies should make use of random sampling methods so that generalization can be enhanced by diversifying samples. Longitudinal follow-up would allow more valuable insights; it would quantify long-term outcomes and the impact of discharge readiness on readmissions and complications. Individualized discharge plans should be developed for infant caregivers and those undergoing elective surgeries to enhance discharge education that meets specific needs. Training health professionals through appropriate training programs will also help provide respectful and professional services to overcome other cultural and language barriers of non-Saudi caregivers. And finally, support networks, including psychological counseling, need to be established to extend support to the caregivers during hospitalization.

## Figures and Tables

**Table 1:** Caregiver and child characteristics (transition conditions).

Demographic Characteristics	*N* = (258)	%
Caregiver Gender		
Female	253	98.1
Male	5	1.9
Caregiver Relationship to Child		
Mother	241	93.4
Father	5	1.9
Sister/Brother	4	1.6
Grandmother/father	2	0.8
Aunt/Uncle mother’s side	2	0.8
Aunt/Uncle father’s side	4	1.6
Caregiver Nationality		
Saudi	178	69.0
Non-Saudi	80	31.0
Caregiver Marital Status		
Married	232	89.9
Single	9	3.5
Widowed	5	1.9
Divorced	12	4.7
Caregiver Educational Level		
Elementary School	21	8.1
Middle School	39	15.1
High School	67	26.0
Bachelor	109	42.2
Higher Education	6	2.3
Illiterate	16	6.2
Child Age		
Infants 0–2	90	34.9
Childhood	141	54.7
Adolescents	27	10.5
Payor		
Cash	16	6.2
Medical insurance	11	4.3
Governmental Org.	199	77.1
Charitable Org.	32	12.4
Caregiver age	Mean ± SD	34.04 ± 7.6
Stay length (Days)	Mean ± SD	7.72 ± 8.5

**Table 2:** Readiness for Hospital Discharge Scale (response patterns).

Readiness Domains	Items	Mean	SD
Personal Status			
a-Parent personal status	How would you describe your strength today?	8.28	2.65
b-Child personal status	How would you describe your child’s strength today?	8.62	2.26
Knowledge	How much do you know about problems to watch for after you go home?	7.49	3.27
	How much do you know about what your child is allowed and not allowed to do after you go home?	8.04	2.98
	Overall	7.76	2.75
Coping	How well will you be able to handle the demands of life at home?	8.53	2.21
	How well will you be able to perform your child’s medical treatments (for example, caring for a wound, breathing treatments, using equipment, or giving medications in the correct amounts and at the correct times) at home?	9.14	1.87
	Overall	8.83	1.66
Expected Support	How much help will you have, if needed, with your child’s personal care after you go home?	7.89	3.18
	How much help will you have, if needed, with household activities (for example, cooking, cleaning, shopping, babysitting) after you go home?	7.49	3.43
	Overall	7.69	2.96

**Table 3:** Quality of Discharge Teaching Scale (QDTS).

Domains	Items	Mean	SD
Content	How much information did you need from your child’s nurses about taking care of your child after you go home?	6.55	3.79
	How much information did you need from your child’s nurses about your emotions after you go home?	6.32	4.03
	How much information did you need from your child’s nurses about your child’s medical needs or treatments (for example, caring for a wound, breathing treatments, using equipment,	6.34	3.82
	How much practice did you need with your child’s medical treatments or medications before going home?	5.37	4.17
	How much information did you need from your child’s nurses about who and when to call if your child has problems after you go home?	5.38	4.18
	How much information did your family member(s) or others need about your child’s care after you go home from the hospital?	5.81	4.03
	Overall	5.96	2.77
Received	How much information did you receive from your child’s nurses about taking care of your child after you go home?	5.79	4.234
	How much information did you receive from your child’s nurses about your emotions after you go home?	5.83	4.214
	How much information did you receive from your child’s nurses about your child’s medical needs or treatments after you go home?	6.33	4.059
	How much practice did you have with your child’s medical treatments or medications before going home?	5.50	4.180
	How much information did you receive from your child’s nurses about who and when to call if your child has problems after you go home?	4.91	4.370
	How much information did your family member(s) or others receive about your child’s care after you go home from the hospital?	5.36	4.220
	Overall	5.62	3.18
Delivery	How much did the information provided by your child’s nurses answer your specific concerns and questions?	6.86	3.64
	How much did your child’s nurses listen to your concerns?	7.08	3.66
	Were your child’s nurse’s sensitive to your personal beliefs and values?	7.95	3.07
	Did you like the way your child’s nurses taught you about how to care for your child at home?	7.11	3.77
	Was the information your child’s nurses provided about caring for your child given to you in a way you could understand?	7.12	3.78
	Did your nurses break up you’re teaching into small amounts to help you learn?	6.84	3.80
	Did your child’s nurses check to make sure you understood the information and instructions?	6.91	3.79
	Did you receive consistent (the same) information from your child’s nurses, doctors, and other health workers?	7.87	3.19
	Was the information about caring for your child given to you at times that were good for you?	7.90	3.21
	Was the information you received from your child’s nurses given at times when your family member(s) or others could attend?	4.67	4.29
	Did your child’s nurses help you to feel confident in your ability to care for your child at home?	6.80	3.95
	How confident do you feel that you would know what to do in an emergency?	7.48	3.24
	Did the information your child’s nurses provided about your child’s care at home decrease your anxiety about going home?	6.69	3.82
	Overall	7.02	2.73

**Table 4:** The *t*-test results for readiness for hospital discharge between two hospitals.

	Hospital Affiliation	Mean ± SD	*t*-Test	Mean Difference	*p*-Value	95% CI
Overall_RDH	MOH	8.35 ± 1.53	1.840	0.347	0.067	−0.02, 0.72
	KAUH	8.01 ± 1.50				

**Table 5:** The *t*-test results for quality of discharge teaching between two hospitals.

	Hospital Affiliation	Mean ± SD	*t*-Test	Mean Difference	*p*-Value	95% CI
Overall_QDT	MOH	5.48 ± 2.89	1.840	−0.948	0.006	−1.62, −0.28
	KAUH	6.43 ± 2.56				

**Table 6:** Multivariate regression model.

Model:	Ind.	Model Summary	B	Std. Error	Standardized B	*t*	Sig.
Model 1: Parent Personal Status	Constant	F (2, 255) = 6.37, *p* = 0.002, R_2_ = 0.05, adjusted R_2_ = 0.04	5.422	0.827		6.560	<0.001
	Caregiver age		0.066	0.021	0.189	3.083	0.002
	Received_QDT		0.106	0.051	0.128	2.080	0.039
Model 2: Child Personal Status	Constant	F (2, 255) = 6.83, *p* = 0.001, R_2_ = 0.05, adjusted R_2_ = 0.043	8.236	0.489		16.829	<0.001
	Child Age		0.736	0.226	0.205	3.256	0.001
	Diagnosis Type		−0.572	0.230	−0.157	−2.490	0.013
Model 3: Coping_RDH	Constant	F (3, 254) = 7.83, *p* < 0.001, R_2_ = 0.09, adjusted R_2_ = 0.07	7.558	0.548		13.785	<0.001
	Stay length		−0.039	0.012	−0.197	−3.238	0.001
	Delivery_QDT		0.089	0.037	0.146	2.426	0.016
	Caregiver age		0.028	0.013	0.127	2.093	0.037
Model 4: Knowledge_RDH	Constant	F (3, 254) = 7.83, *p* < 0.001, R_2_ = 0.09, adjusted R_2_ = 0.07	7.512	0.378		19.852	<0.001
	Stay length		−0.047	0.020	−0.144	−2.351	0.019
	Received_QDT		0.109	0.053	0.126	2.055	0.041
Model 5: Expected_RDH	Constant	F (1, 256) = 12.17, *p* = < 0.001, R_2_ = 0.05, adjusted R_2_ = 0.04	9.475	0.543		17.461	<0.001
	Caregiver Nationality		−1.362	0.391	−0.213	−3.488	<0.001

## Data Availability

The data presented in this study are available upon request from the corresponding author. The data are not publicly available due to information that could compromise the privacy of the research participants.
